# Native-State Stability Determines the Extent of Degradation Relative to Secretion of Protein Variants from *Pichia pastoris*

**DOI:** 10.1371/journal.pone.0022692

**Published:** 2011-07-27

**Authors:** Graham Whyteside, Marcos J. C. Alcocer, Janet R. Kumita, Christopher M. Dobson, Maria Lazarou, Richard J. Pleass, David B. Archer

**Affiliations:** 1 School of Biology, University of Nottingham, Nottingham, United Kingdom; 2 School of Biosciences, University of Nottingham, Sutton Bonington, United Kingdom; 3 Department of Chemistry, University of Cambridge, Cambridge, United Kingdom; 4 Liverpool School of Tropical Medicine, Liverpool, United Kingdom; Emory University School of Medicine, United States of America

## Abstract

We have investigated the relationship between the stability and secreted yield of a series of mutational variants of human lysozyme (HuL) in *Pichia pastoris*. We show that genes directly involved in the unfolded protein response (UPR), ER-associated degradation (ERAD) and ER-phagy are transcriptionally up-regulated more quickly and to higher levels in response to expression of more highly-destabilised HuL variants and those variants are secreted to lower yield. We also show that the less stable variants are retained within the cell and may also be targeted for degradation. To explore the relationship between stability and secretion further, two different single-chain-variable-fragment (scFv) antibodies were also expressed in *P. pastoris,* but only one of the scFvs gave rise to secreted protein. The non-secreted scFv was detected within the cell and the UPR indicators were pronounced, as they were for the poorly-secreted HuL variants. The non-secreted scFv was modified by changing either the framework regions or the linker to improve the predicted stability of the scFv and secretion was then achieved and the levels of UPR indicators were lowered Our data support the hypothesis that less stable proteins are targeted for degradation over secretion and that this accounts for the decrease in the yields observed. We discuss the secretion of proteins in relation to lysozyme amyloidosis, in particular, and optimised protein secretion, in general.

## Introduction

Yeasts have become increasingly common hosts for the expression of eukaryotic heterologous proteins due to their ease of culture and genetic manipulation, well defined fermentation processes and rapid growth to high cell densities. These advantages have led to a number of studies concerning the optimisation of yeast as cell factories for the secretion of heterologous proteins that include therapeutic proteins [1], [2]. The original yeast system used for heterologous protein secretion was the baker's yeast *Saccharomyces cerevisiae*, and its most notable successes have been the production of insulin and hepatitis B surface antigen [3], [4]. More recently, the methylotrophic yeast *Pichia pastoris* has become a popular expression host. *P. pastoris* has many advantages over *S. cerevisiae* including growing to higher cell densities, the availability of strong and tightly controlled promoters and having a low immunogenic glycosylation pattern [5]. These advantages combined with the recently published genome sequence [6], [7] of this organism have made *P. pastoris* the yeast expression system of choice for many researchers.

Over-expression of heterologous proteins in yeasts has been shown to exceed the folding capacity of the ER and activate the unfolded protein response [8]. The activation of the UPR affects the transcription of ∼400 genes in yeasts and filamentous fungi [9]–[11]. The majority of transcriptionally-affected genes encode for proteins associated with protein folding and secretion as well as proteolysis via ERAD [8], [9]. Therefore the activation of the UPR is an attempt by the cell to alleviate the stress on the ER by not only increasing the folding capacity of the ER, but also by removing mis-folded/unfolded proteins for degradation. In *S. cerevisiae*, activation of the UPR is mediated through the ER membrane protein Ire1p which detects the presence of unfolded proteins via its lumenal amino terminal domain [12], [13]. Once Ire1p has detected unfolded proteins within the ER, it oligomerises and trans-autophosphorylates through its cytosolic kinase domain [14], [15]. Trans-autophosphorylation stimulates the activity of the Ire1p C-terminal domain which cleaves the mRNA of its only known substrate *HAC1^u^* (unspliced) and this splicing event removes a non-conventional intron from the *HAC1^u^* mRNA to yield the translationally competent HAC1^i^ (spliced) mRNA [16], [17]. *HAC1^i^* mRNA is then efficiently translated to create the transcription factor Hac1p. Once translated, Hac1p activates target gene transcription by binding to a specific upstream sequence termed the unfolded protein response element (UPRE) [18]. Many of these target genes are involved in aspects of protein folding and secretion and include encoded chaperones, foldases and genes involved in ERAD [8], [9]. Furthermore, continual ER stress is linked to activation of ER-phagy which is an ER-specific form of autophagy where parts of the ER containing terminally mis-folded proteins are transported to the vacuole for degradation [12]. In this study we have assessed the activation of these pathways by over-expressing mutational variants of the human lysozyme protein (HuL) which differ in their native-state stabilities. We have previously shown that the final secreted yields of the HuL variants from *P. pastoris* are dependent on the stability of the variant, with the higher native-state stability resulting in greater secretion levels [19]. Furthermore, this effect was independent of mRNA levels and is therefore post-translational indicating that these constructs will provide useful insights into the way highly similar proteins are assessed and folded by the ER. The secretory levels of HuL variants are of great interest as a number of mutational variants have been linked with systemic amyloidosis in which large amounts of the variants accumulate extracellularly in the form of intractable fibrillar deposits [20]. In the study presented here we have used these highly-similar variant HuL proteins, that differ in stability, to assess the changes in transcription levels of genes from the UPR, ERAD and ER-phagy via qRT-PCR. This analysis provides, for the first time, a clear correlation between the expression of genes involved in the folding and secretory apparatus within cells and the folded-state stability of an extracellular protein. The study shows that the cell is highly sensitized to detect and then respond to proteins of particular stabilities. The relevance of our findings to lysozyme amyloidosis is discussed and we also use the information in an application of biotechnological relevance by devising a strategy for engineering the secretion of a scFv from *P. pastoris*.

## Results

### UPR genes are activated upon expression of the human lysozyme variants

**Figure 1:**
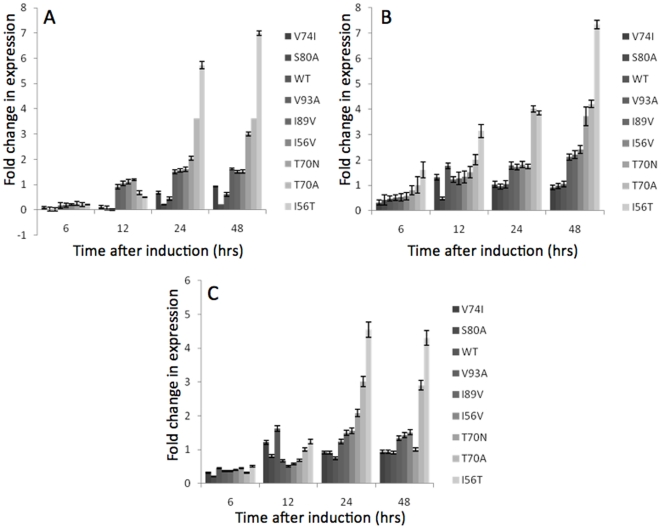
Changes in gene expression for elements of the UPR upon induction of expression of the HuL variants (Grayscales: from dark to light  =  from most stable to least stable). Analysis of mRNA levels was carried out by qRT-PCR. RNA levels were plotted as fold increase above those RNA levels from time zero and are plotted with respect to time (see Methods). All values were normalised to levels of actin transcription and represent the average of at least 3 independent experiments, with the error bars representing standard deviation. **A** *HAC1*, **B** *KAR2*, **C** *PDI1*.

We have previously reported that the secreted yields of the single amino acid HuL variants were independent of mRNA levels [19]. The over-expression of heterologous proteins can overwhelm the capacity of the ER to assist the folding and secretion of proteins which, in turn, leads to activation of the UPR. In addition, the UPR can also be activated by accumulation of mis-folded/unfolded proteins within the ER via the Kar2p/Ire1p detector system [12]. It was hypothesised [19] that the reason for the decrease in secretion of HuL variants demonstrating reduced stability was due to mis-folding or decreased folding efficiency of the variants. If this hypothesis were to be correct the unfolded/folded equilibrium of the protein within the ER would be shifted towards the accumulation of mis-folded proteins and therefore lead to activation of the UPR. To test this hypothesis the nine HuL variants were expressed in *P. pastoris* for 48h and the mRNA levels of the UPR marker genes *HAC1, KAR2* and *PDI1* were assessed at 6, 12, 24 and 48 h using qRT-PCR. Figure 1 shows that all of the genes follow a general trend of higher mRNA expression levels for the least stable HuL variants as compared to the more stable constructs. In the case of *HAC1* transcript level (Figure 1A), expression of the least stable HuL variant, I56T, leads to a pronounced increase of *HAC1* mRNA throughout the experiment with a 6-fold increase in transcript levels at 48 h post-induction. In contrast, cells expressing the most stable HuL variants, wild-type (WT), S80A and V74I, showed relatively small increases in *HAC1* expression with a less than 1-fold increase in mRNA levels for all three variants. Overall the data shown in Figure 1A revealed that the expression level of *HAC1* is linked to the stability of the HuL variant expressed, with the least stable variants stimulating a large and pronounced response whereas increased stability decreased the need for the transcription of *HAC1*. As described previously the *HAC1* gene encodes a transcription factor which controls the activation of the UPR [21]. It is therefore logical to postulate that the variant-dependent increase in levels of *HAC1* mRNA observed in Figure 1A would lead to an increase in UPR activation. It has been described previously that activation of the UPR is characterised by an increase in the transcription levels of a number of genes that are directly affected by Hac1p [9]. These genes include the molecular chaperone *KAR2* and the protein disulfide isomerase *PDI1.* To test whether the up-regulation of *HAC1* transcript levels correlates to an increase in UPR, the mRNA levels of these two genes were assessed. Figures 1B and C show the levels of *KAR2* and *PDI1* in response to expression of the HuL variants and confirm that the UPR is indeed activated in all of the strains with the level of activation being dependent on variant stability. Expression of the least stable I56T variant shows the highest level of gene expression with *KAR2* up-regulated by ∼7.5-fold and *PDI1* 5-fold after 48 h, whereas the most stable variants WT, S80A and V74I all have much lower levels of expression, at up to 9-fold less than I56T (Figure 1B and 1C). Indeed these data clearly demonstrate that there is a strong correlation between the gene expression levels, and therefore UPR activation, and HuL stability.

### ERAD activation upon expression of the human lysozyme variants

**Figure 2:**
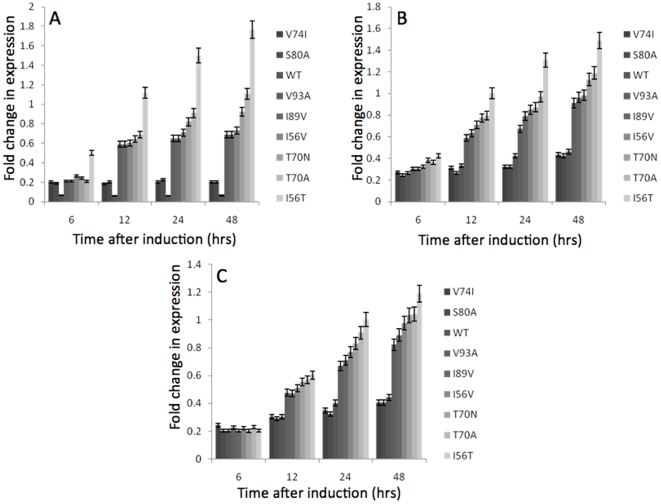
Changes in gene expression components of ERAD upon induction of expression of the HuL variants (Grayscales: from dark to light  =  from most stable to least stable). Analysis of mRNA levels was carried out by qRT-PCR. RNA levels were plotted as fold increase above those RNA levels from time zero and were plotted with respect to time (see Methods). All values were normalised to the levels of actin transcription and represent the average of at least 3 independent experiments, with the error bars representing standard deviation. **A** *DER1*, **B** *HRD3*, **C** *SEC61*.

In addition to UPR activation, accumulation of mis-folded/unfolded proteins in the ER activates ERAD, another process aimed at alleviating ER stress, which involves the retro-translocation of unfolded/mis-folded polypeptides from the ER to the cytoplasm for subsequent degradation. ERAD has also been shown to be functionally linked to the UPR with certain genes essential for ERAD, such as *HRD3*, *DER1* and *SEC61*, up-regulated in an *IRE1*-dependent manner [9]. To assess if expression of the HuL variants has any effect on the transcription of these genes, thereby triggering ERAD, qRT-PCR analysis of the mRNA levels of *DER1*, *HRD3* and *SEC61* was monitored (Figure 2A, 2B and 2C, respectively). Transcription of each of these genes was up-regulated in a variant-specific manner with the least stable variant, I56T, stimulating the highest level of gene expression with increases of, 1.8-fold for *DER1,* 1.6-fold for *HRD3* and 1.2-fold for *SEC61*. In addition, the most stable variant, V74I, shows only slight up-regulation of the mRNA levels of each of the genes, 0.2-fold for *DER1*, 0.4-fold for *HRD3* and 0.4-fold for *SEC61*. A distinct relationship between the gene expression levels and the stability of the HuL variants was again observed, in a similar fashion to the results observed with the up-regulation of the genes related to UPR activation, suggesting that the native-state stability of the protein is a major determining factor in the activation of both ERAD and UPR activity. This predicted activation of ERAD in a stability-dependent manner suggests that proteins with decreased stability, not only activated the UPR, but are also targeted for degradation as opposed to being folded correctly and secreted. The predicted up-regulation of ERAD in this way indicates that the folding environment of the ER is very sensitive to changes in the folding equilibrium of a protein and even small changes in this equilibrium may have important consequences for the final secreted yields of the target proteins.

### Autophagy activation upon expression of the HuL variants

**Figure 3:**
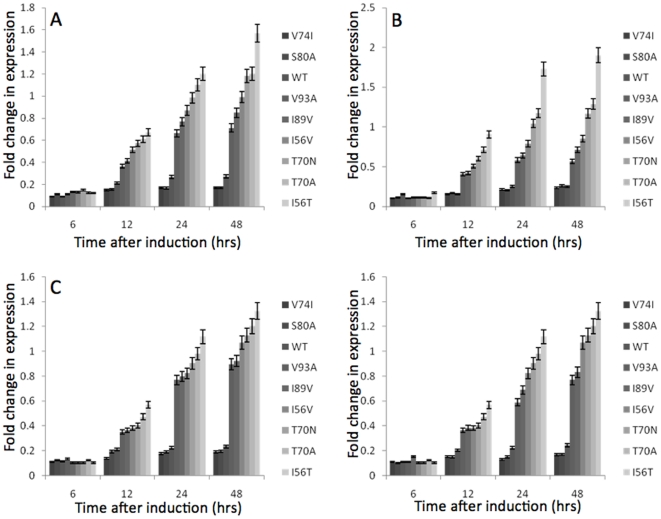
Changes in gene expression for components of ER-phagy upon induction of expression of the HuL variants (Grayscales: from dark to light  =  from most stable to least stable). Analysis of mRNA levels was carried out by qRT-PCR. RNA levels are plotted as fold increase above the RNA levels from time zero and were plotted with respect to time (see Methods). All values were normalised to the levels of actin transcription and represent the average of at least 3 independent experiments, with the error bars representing standard deviation. **A** *ATG8*, **B** *ATG1*, **C** *ATG9*, **D** *ATG16,*

Severe ER stress causes prolonged activation of the UPR and up-regulates a process termed ER-phagy [22]. This process serves to remove sections of the ER in an attempt to relieve ER stress and is involved in the clearance of mis-folded proteins from the ER [22], [23]. The transcript levels of the genes *ATG8, ATG1, ATG9* and *ATG16,* each of which is involved in ER-phagy, were assessed during expression of the HuL variants (Figure 3A, 3B, 3C and 3D). The highest level of gene expression observed is, in common with the UPR and ERAD studies, for the least stable HuL variant, I56T, with the levels of ER-phagy transcripts being 6–9-fold higher than the most stable variant, V74I. The increase in gene expression for the three most stable HuL variants is very low; ∼0.1-fold across all the ER-phagy genes analysed and indicates that these variants are less likely to mis-fold and result in an ER stress response. These data indicate that the least stable HuL variants are causing severe stress on the ER, and this cannot be dealt with via an increased folding capacity (UPR) or degradation (ERAD); however, a formal direct demonstration of ER-phagy induction would provide further confidence in this model.

### Intracellular levels of the HuL variants

**Figure 4:**
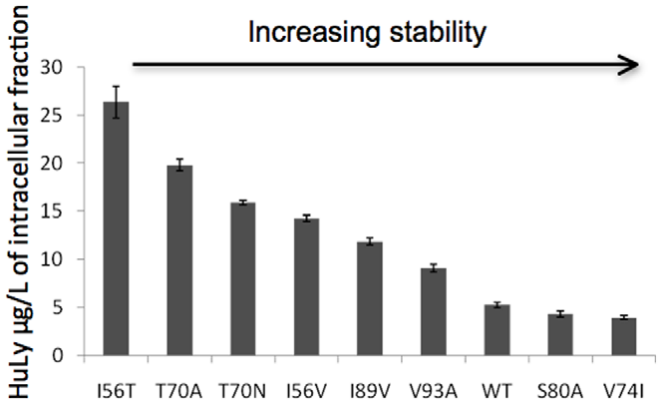
Intracellular levels of HuL variants expressed in *P. pastoris* as measured by ELISA. Each value represents that mean of 3 independent experiments and the error bars represent the standard deviation.

The levels of UPR, ERAD and ER-phagy activation are directly related to the native-state stability and therefore to the amounts of the HuL variant that are secreted. To determine whether this was due to increased intracellular retention of the least stable HuL variants, intracellular fractions of *P. pastoris* strains expressing the HuL variants, were analysed for lysozyme content via ELISA. Figure 4 shows the intracellular levels of the HuL variants 48 h after induction. The levels of intracellular retention increased with decreasing HuL native-state stability; the yeast expressing the least stable variant contained five times more lysozyme protein within the cells than the yeast expressing the most stable variant. Furthermore, the data show a direct relationship between the intracellular retention of HuL and the stability of the HuL variant with a stepwise reduction in intracellular retention as stability increases.

### Secretion of scFvs from *P. pastoris*

**Figure 5:**
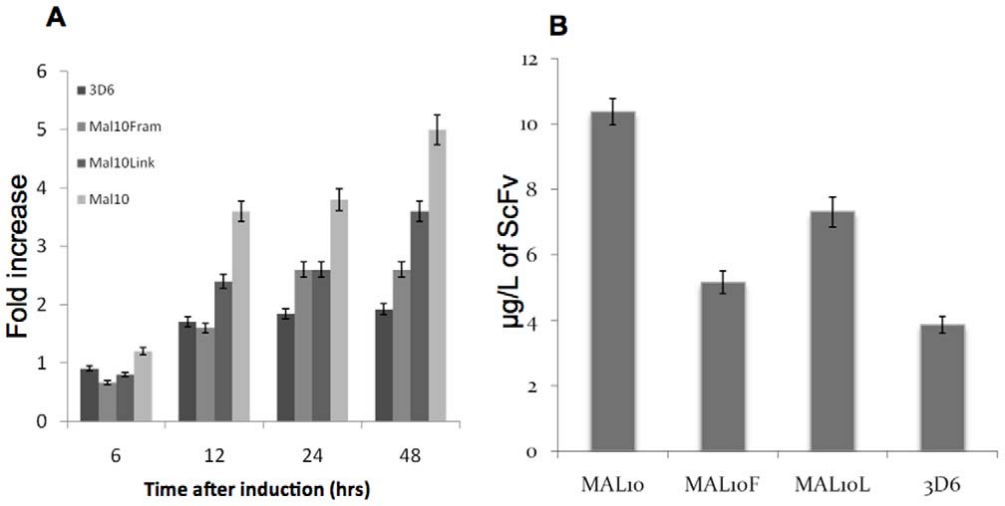
Induction of scFvs and impact on *KAR2* mRNA levels. **A** Changes in gene expression of *KAR2* upon induction of 3D6, Mal10, Mal10Fram and Mal10Link. Analysis of mRNA levels was carried out by qRT-PCR. RNA levels were plotted as fold increase above those RNA levels from time zero and are plotted with respect to time (see Methods). All values were normalised to levels of actin transcription and represent the average of at least 3 independent experiments, with the error bars representing standard deviation. **B** Intracellular levels of the scFv variants expressed in *P. pastoris* as measured by ELISA. Each value represents that mean of 3 independent experiments and the error bars represent the standard deviation.

**Figure 6:**
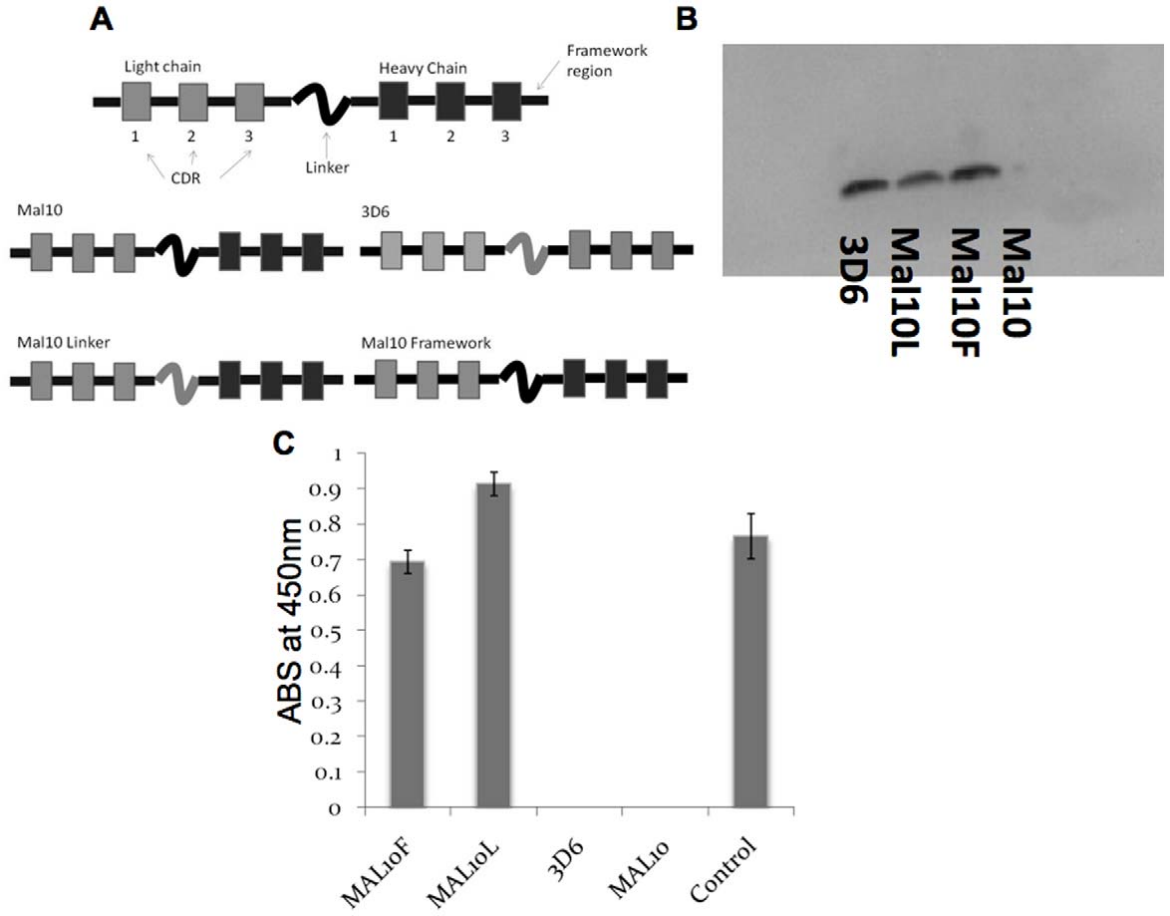
Description and detection of antibodies. **A** Schematic diagram of Mal10, 3D6, Mal10Fram and Mal10link scFvs used in this study. **B** Western blot analysis of the secretome from *P. pastoris* strains expressing Mal10, 3D6, Mal10Fram and Mal10Link. All scFvs were designed with a C-terminal hexahistidine tag to allow detection using an anti-His Ab. **C** ELISA for recognition of the Mal10 antigen MSP1_19_-GST by Mal10, 3D6, Mal10Fram, Mal10Link and the control monoclonal Ab. ABS is absorbance. Each bar represents the mean of 3 experiments and the error bars represent the standard deviation.

The data presented in the previous section show that there is a strong correlation between protein stability, final secreted yield and activation of ER stress responses. We then examined the expression of two scFvs: a non-secreting scFv (Mal10) was compared to a secreting scFv (3D6) and we assessed the transcript level of *KAR2*, a UPR marker gene, in those two *P. pastoris* strains. The level of *KAR2* mRNA is higher in the Mal10 strain as compared with 3D6 (Figure 5A). Alignment of both the scFv sequences showed significant variation between the proteins in both the heavy (42 differences) and light chains (21 differences). The linkers also differed: GGGGSGGGGSGGGS (3D6) and GSTSGSGKPGSGEGSTKG (Mal10). Variants of the Mal10 scFv were constructed in which either the framework regions of Mal10 were replaced by those from 3D6 (Mal10Fram) or the linker of Mal10 was replaced by the 3D6 linker (Mal10Link) (Figure 6A). All scFvs were expressed at similar mRNA levels (Figure S1). The levels of *KAR2* transcript are higher in the Mal10, non-secreting scFv strain than in 3D6, Mal10Link or Mal10Fram variants (Figure 5A). In contrast to Mal10, the Mal10Fram and Mal10Link scFv variants are secreted from *P. pastoris* (Figure 6B) at levels between 1 and 5 mg/L in un-optimised shake flask cultures. Mal10 is detected at higher levels within the *P. pastoris* cells than the secreted scFvs (Figure 5B). The Mal10 scFv was predicted [24] to be less stable than 3D6 and the Mal10Fram scFv was predicted to be more stable than Mal10 by 30.15 Kcal/mol. The Mal10Fram and Mal10Link scFvs were shown by ELISA to recognise the cognate antigen (Figure 6C) indicating that the sequence changes made in order to afford secretion did not alter antigen recognition and binding.

## Discussion

In our previous study we showed that the secreted yield of HuL was dependent on its stability, with the least stable variants secreted to a lesser degree than more stable mutants [19]. Despite the fact that the intrinsic native-state stability of the HuL variants examined in this study require temperatures of ∼65–80°C for 50% of the molecules to be unfolded [19], we have observed clear effects of the least stable HuL variants on the up-regulation of degradation processes, suggesting that in normal organisms, these quality control mechanisms are quite stringent. In this study we have aimed to understand the cellular processes involved and to apply this knowledge to formulate an approach to improve scFv secretion. Initially, we investigated the effect that expression of the HuL variants in *P. pastoris* has on activation of the UPR. This was achieved by using qRT-PCR to measure the change in transcription of *HAC1*, *KAR2* and *PDI1*. The levels of *HAC1* transcript were seen to increase in a variant-dependent manner with the least stable HuL variants activating a larger transcriptional (6-fold) response than more stable variants (1-fold). This transcriptional up-regulation of *HAC1* indicates that the UPR is activated to a much higher level in strains expressing less stable variants and is indicative of a higher ER stress in these cells. These findings are in good agreement with observed UPR activation in a *Drosophila* model expressing the amyloidogenic lysozyme variants (F57I and D67H). In this case, direct detection of the Ire-1 pathway of UPR activation is monitored by an xbp1-EGFP marker; splicing of the transgenic xbp1-EGFP-mRNA by Ire-1 brings the EGFP coding sequence into frame, generating GFP fluorescence (Kumita, Brorsson et al., unpublished data). The up-regulation of *HAC1* mRNA has also been reported in *S. cerevisiae* [25], although it has previously been reported that the levels of *HAC1* mRNA in *S. cerevisiae* remain relatively stable until induction of the super-UPR (S-UPR) [26]. Activation of the S-UPR in *S. cerevisiae* is only ever observed upon a dual stressing of the ER environment, e.g. DTT and tunicamycin treatment. Our data suggest that *P. pastoris* is able to activate the UPR to a higher level more quickly than in *S. cerevisiae* and may have an increased ability to fold proteins within the ER. This factor may be an underlying reason for the relatively higher secreted production of heterologous proteins from *P. pastoris.* The variant-dependent increase in the UPR activating Hac1p transcription factor was mirrored by an increase in the transcript levels of *KAR2* and *PDI1*. The increases were variant-dependent with the least stable variants increasing mRNA levels to a greater level, 5–9 fold, than more stable variants. Thus, the least stable HuL variants are causing more ER stress and therefore activating the UPR to a greater degree.

In addition to analysing the response of the UPR to the expression of HuL variants with differing thermostabilities the activation of other ER stress control mechanisms was measured at the level of gene expression. The activation of genes involved in ERAD and ER-phagy was assessed during expression of the HuL variants via qRT-PCR of the transcript levels of the selected genes essential for these processes. All of these genes were activated in a variant-specific manner with the least stable variants increasing transcription of the genes essential for these cellular functions to a higher level than in strains expressing more stable variants. These data strongly suggest that the unstable HuL variants are terminally misfolding within the ER and therefore activating the various homeostatic mechanisms in an attempt to alleviate the stress on the ER. However, as these proteins are unstable the increase in folding capacity provided by the UPR is not enough to clear the accumulation of mis-folded proteins and therefore the degradative processes of ERAD and ER-phagy are activated. The hypothesised accumulation of proteins within the cells (and possibly within the ER) was assessed via quantitative analysis of the intracellular fractions of *P. pastoris* strains expressing the HuL variants using ELISA. This study showed that the least stable HuL variants were being retained within the cell to a greater extent than the more stable variants. Accumulation of these least stable variants could then account for the higher expressed levels of genes involved in the UPR, ERAD, and ER-phagy. Even though each HuL variant differs by just one amino acid from the WT, the cells are able to distinguish each variant, indicating sensitivity to protein folding within the ER. The HuL variants provide a series of proteins that probe the sensitivity of cellular responses to folding.

Previously it has been observed that the degree of destabilisation of HuL variants correlates with the propensity of the protein to convert from its normal soluble state into intractable amyloid fibrils, as well as with the degree which the protein is degraded [20], [27]. This may suggest that in patients suffering from amyloidosis the destabilised nature of the variant lysozyme is of great importance to protein misfolding. We have demonstrated here that in a normally functional organism, the ER quality control mechanisms suppress the secretion of destabilised lysozyme variants, including the I56T variant associated with amyloid disease. Indeed, a similar correlation has been observed in *Drosophila* models expressing lysozyme variants (Kumita, Brorsson *et al*., unpublished information). At first, these findings appear to be in contradiction to the fact that in patients suffering from lysozyme amyloidosis, the large quantities of fibrillar aggregates which accumulate appear to be comprised solely of the destabilised lysozyme variants [28]. Possibly, the destabilised variants are able to be secreted in sufficient quantities into the extracellular space and eventually aggregate in these patients. As these variants are relatively stable proteins, our data may suggest that ability of the quality control processes are somehow overwhelmed in these patients and may lead to a possible mechanism by which these amyloidogenic variants interface with, and possibly elude, the quality control machinery, thereby leading to the manifestation of disease. We do not yet have a full mechanistic understanding of how the cell perceives the lysozyme variants differently and then responds. We did not examine if lysozyme fibrils accumulate in the ER of *P. pastoris* but there was no evidence for that happening in the ER of *Drosophila* when expressing I56T (Kumita, Brorsson *et al*., unpublished information). Nor does it follow that a decrease in the folded-state stability of a variant necessarily slows its rate of folding and, thereby, alter the balance between folded and unfolded forms. The I56T lysozyme variant has previously been shown, *in vitro*, to form its native state two-fold faster than the WT lysozyme, despite the folded state of I56T being less stable [29].

This study also provides a systematic approach for engineering increased secretion of target proteins. The impact of folded state stability of HuL variants on their potential for secretion suggests that the stability of a protein could be used as an indicator of secretion potential. That hypothesis will be an over-simplification for the secretion of many proteins but folded state stability should be considered when formulating a predictive model for secretability. This knowledge was applied in an examination of the secreted yields of a series of variant scFvs. Two scFvs were examined initially where one was secreted but the other was not. We predicted their respective stabilities by using an amino acid sequence consensus-based approach and measured the mRNA levels of indicator genes for ER status. The predictions indicated that the secreted scFv (3D6) was likely to be more stable than the non-secreted scFv (Mal10) and the *KAR2* mRNA level was higher when expressing Mal10. We then engineered Mal10 in two ways. Firstly, the framework regions of Mal10 were replaced by those from 3D6 and, secondly, the Mal10 linker was replaced by the glycine-serine repeat linker from 3D6. In both cases, the Mal10Fram and Mal10Link scFvs were predicted to be more stable than Mal10 and both were secreted. Furthermore, in line with a prediction of the impact of stability on the UPR, Mal10 induced the UPR to a higher extent than the other, more stable, scFv variants. While we acknowledge that engineering scFvs in this way will introduce unknown effects on folding and stability, the study serves to illustrate an approach that can be used to predict secreted yields.

In conclusion, the folded-state stabilities of HuL variants determine their secreted yields. The yields are probably a consequence of ER homeostasis being disturbed by even small changes in folded-state stability of the target protein. The ER stress induces a variety of responses that aim at restoring cellular homeostasis. The secretion of scFvs can be improved by engineering the scFv for increased secretion based on prediction of their stabilities.

## Materials and Methods

### Materials

All restriction enzymes and Phusion polymerase were purchased from New England Biolabs Ltd (Hitchin, UK). Synthetic oligonucleotides and chemicals were purchased from Sigma-Aldrich (Gillingham, UK).

### Strains and plasmids

*P. pastoris* GS115 (Invitrogen) was used as the host strain for expression of the human lysozyme variants [19] and the scFv constructs. The human lysozyme variants were expressed from a pPIC9-based plasmid under the control of the methanol-inducible *AOX1* promoter and were directed for secretion by fusion to the α-factor secretion signal. The scFvs were expressed from the pPICZαA vector as per manufacturer's instructions (Invitrogen).

### HuL variants and scFvs

The construction of HuL variant HuL has been described previously [17]. The Mal 12.10 scFv (described below as Mal10) is directed against *Plasmodium falciparum* merozoite surface protein 1 (PfMSP1-19) [30], [31]. The 3D6 scFv was obtained from Diethard Mattanovich (BOKU, Vienna) and was derived from the cell line 3D6 and derived human monoclonal antibody against an HIV protein, HIV-1-gp41 [32]. All scFvs included a C-terminal hexaHis tag for detection.

### Expression of human lysozyme variants and scFvs

A single colony from each variant was inoculated into Buffered Minimal Glycerol media (BMG) (100 mM potassium phosphate, pH 6.0, 1.34% (w/v) YNB, 4×10^−5^% w/v biotin, 1% w/v glycerol) and incubated for 2 4h (30°C, 230 rpm). The samples were the centrifuged (5000 g, 4°C, 15 min) and the supernatant discarded. The pellets were then resuspended in 50 ml of Buffered Minimal Methanol (BMM) media (as BMG but 0.5% v/v methanol in place of glycerol) and protein expression was induced for 48 hrs with 0.5% methanol being replenished every 24 hrs. After 48 hrs the OD_600nm_ of the culture was measured and the secreted yield of human lysozyme was assayed as described [19]. The data were normalised using the procedure described in [19]. Yields of scFvs were quantified using ELISA (below).

### RNA extraction and cDNA synthesis

RNA extraction was performed as described [33]. cDNA was synthesised from 1 µg of purified RNA using Superscript III (Invitrogen) according to the manufacturer's instructions.

### qRT-PCR

qRT-PCR was carried out on cDNA synthesised from total mRNA. Primer and probe sets were designed using Primer Express 3 (Applied Biosystems) and synthesised at Sigma-Aldrich (Gillingham, UK) using published genome sequences [6], [7]. Probes were synthesised with TAMRA and FAM probes for use in the Taqman method of qRT-PCR. All samples were compared to the internal standard actin and relative quantities were determined in relation to this control. Actin and *PGK1* showed stable gene expression during the course of the experiments (Figures S2A and S2B) and *PGK1*, a gene not involved in protein folding, was used as a control for general gene expression (Figure S2B). Standard curves were constructed from pooled cDNA samples. The reaction was carried out using Applied Biosystems fast-universal master mix and the reactions were designed using the Applied Biosystems 7500 fast real-time PCR software. Data are presented as fold increase above the basic control value (i.e. compared to the values at the zero time point). Thus, no change from the control value is recorded as zero and a doubling in gene expression level is plotted as a value of 1. Primer and probe sets are detailed in the Table S1.

### ELISA of HuL protein

Intracellular retention of the HuL variants was measured using ELISA. Intracellular fractions of *P. pastoris* cells expressing the HuL variants were obtained by following the instructions within the *P. pastoris* manual (Invitrogen). 96 well plates (Maxisorp plates NUNC) were coated with 75 µg/ml of HuL (Sigma), dissolved in phosphate buffered saline (PBS : 137 mM NaCl, 2.7 mM KCl, 10 mM Na_2_HPO_4_, 1.76 mM KH_2_PO_4_ pH 7.4) by incubating at 4°C overnight. The plate was then washed 3 times briefly with PBS-Tween (PBS-T, as PBS with 0.05% v/v Tween 20) and blocked using 3% BSA dissolved in PBS-T (1 h at 37°C). The wells were then washed 3 times briefly with PBS-T. The primary rabbit anti-HuL antibody (Ab) [34] was diluted 1∶40,000 in PBS-T plus 1% BSA and incubated with 1 mg of the intracellular fraction from empty GS115 cells (1–2 h at 37°C). 1 mg of intracellular fraction from HuL-expressing *P. pastoris* was then added and this mixture was placed into the appropriate wells and incubated at 37°C for 1–2 h. After incubation the wells were briefly washed (3x) with PBS-T and incubated with an anti-rabbit horse radish peroxidase-conjugated secondary Ab (Sigma-Aldrich) (diluted 1∶1000 in PBS-T) (1–2 h 37°C). Finally, the plate was washed with PBS-T plus 1% BSA (3 times, 10 minutes each). Sample detection was performed by incubating the wells with 3, 3′, 5, 5′ tetramethylbenzidine (100 µl/well) (Sigma-Aldrich). After 10–20 minutes, the reaction was stopped with the addition of 2 M sulphuric acid (50 µl/well) and the absorbance was measured at 450 nm. Sample readings were compared to a standard curve based on known amounts of purified human lysozyme to quantify the amounts of lysozyme present in each intracellular fraction.

### ELISA of scFv specificity

ELISA for the Mal10 scFv was performed as described above with the following differences. Plates were coated with 5 µg/mL of the control antigen (Ag), recombinant *Pf*MSP1_19_-glutathione-S-transferase, in sodium carbonate (50 mM pH 9) buffer [30]. Supernatant from the culture of scFv-expressing *P. pastoris* strains was applied directly to the Ag-coated plate and detected using a mouse anti-His Ab (Sigma) diluted 1∶20,000. The secondary Ab used was an anti-mouse-HRP conjugated IgG (Sigma) diluted 1∶10,000. An antibody referred to as ‘control’ was the original monoclonal antibody raised against *Pf*MSP1 [30], [31].

### ELISA of scFv amount in cell extracts

Intracellular fractions of *P. pastoris* cells expressing the scFv variants were obtained by following the instructions within the *P. pastoris* manual (Invitrogen). 96 well plates were coated with a range of concentrations (10 to 500 µg/L) of control Ab or 1 mg of intracellular fraction by incubating at 4°C overnight. The scFvs were then detected using a mouse anti-His Ab (Sigma) and an anti-mouse HRP-conjugated secondary Ab (Sigma), both antibodies diluted as above. The procedure was as described above. The amount of scFv was calculated from a standard curve generated by a known amount of Ab adhered to the plate.

### Western blotting

Western blotting was carried out using the Novex XCellLock system (Invitrogen). 20 µg of total protein was added to each well of a pre-cast 12% acrylamide w/v Tris-glycine SDS gel (Invitrogen). Proteins were blotted onto a PVDF membrane using tris-glycine transfer buffer (25 mM Tris; 192 mM glycine; 10% v/v methanol) using electroblotting. The PVDF membrane was then blocked for 1–2 hours in 5% v/v milk in Tris buffered saline-Tween 20, TBS-T (Tris 100 mM pH 7.5; 150 mM NaCl; 0.1% v/v Tween 20). The blot was then washed briefly in TBS-T three times. ScFvs were detected using a mouse anti-His Ab (Sigma) diluted 1∶5,000 in TBS-T plus 0.1% v/v milk by incubating with the membrane for 1 hour at room temperature. After this incubation the membrane was washed three times briefly with TBS-T. The anti-mouse-HRP conjugated secondary Ab was then incubated with the membrane for 1 hour (1∶1000 dilution in TBS-T plus 0.1% v/v milk). The membrane was then washed three times for 10 minutes in TBS-T and detection was performed using 0.1 ml/cm^2^ of 3, 3′, 5, 5′ tetramethylbenzidine. Blots were visualised using a BioRad ChemiDoc imager.

### Engineering scFv for secretion

The complementary determining regions (CDR) of the scFvs were determined using Chothia's numbering [35]. The frequencies for each amino acid and consensus sequence for the scFv was obtained from the abysis database (http://www.bioinf.org.uk/abysis/search.html). The sequences of the Mal10, 3D6 and the consensus sequence, restricted to the framework regions, were compared and the differences collated. The effect of a residue change in the framework region of the scFv was calculated in accordance with Boltzman's law using the equation ΔΔG_th_ =  -RTln(f_o_/f_m_) where f_o_ is the frequency of the original residue at the corresponding position in the original scFv, f_m_ is the frequency of the mutated residue, R is the gas constant, and T is temperature (Kelvin). The resulting changes in free energy were collated using summation as described [24] to predict stabilities, as described elsewhere [36], [37]. Constructs were designed by using DNA2.0 (https://www.dna20.com/) and were cloned into pPICZαA.

## Supporting Information

Figure S1 **Expression of genes encoding scFvs and actin.** **A** Transcript levels measured by qRT-PCR of the scFvs during expression. Each bar represents the mean of 3 independent experiments and the error bars represent the standard deviation **B** Levels of actin expression during expression of the scFv variants.
(TIF)

Figure S2 **Expression from the action gene and** ***PGK1*** **during HuL expression.** **A** Transcript levels of actin during the expression of the HuL variants. Each bar represents the mean of 3 independent experiments and the error bars represent the standard deviation. **B** Gene expression levels of *PGK1* during expression of the HuL variants. All data were normalised to the level of actin transcription and each bar represents the mean of 3 independent experiments with the standard deviation shown as error bars. Expression levels were measured by qRT-PCR and given as artificial units of fluorescence (AU).
(TIF)

Table S1 Primers and probes.
(DOC)
