# Supplementary figures and images for: Native-State Stability Determines the Extent of Degradation Relative to Secretion of Protein Variants from Pichia pastoris
Source: PLoS One. 2011 Jul 27;6(7):e22692. doi: 10.1371/journal.pone.0022692 (PMC3144928; doi:10.1371/journal.pone.0022692)

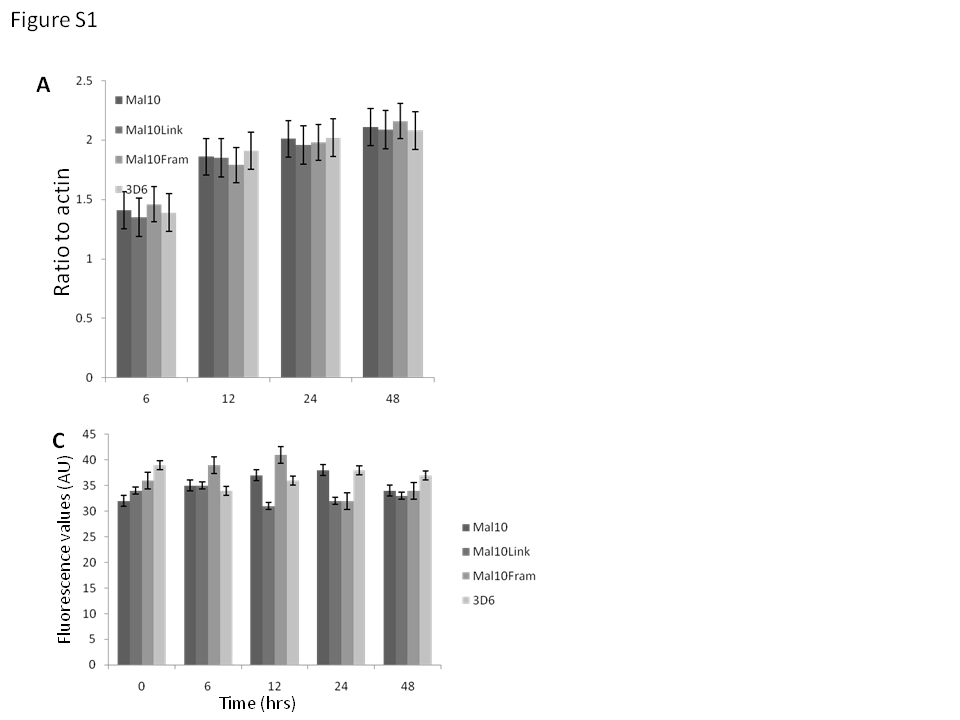

Supplement: Figure S1 — Expression of genes encoding scFvs and actin. A Transcript levels measured by qRT-PCR of the scFvs during expression. Each bar represents the mean of 3 independent experiments and the error bars represent the standard deviation B Levels of actin expression during expression of the scFv variants. (TIF) [file pone.0022692.s001.tif]

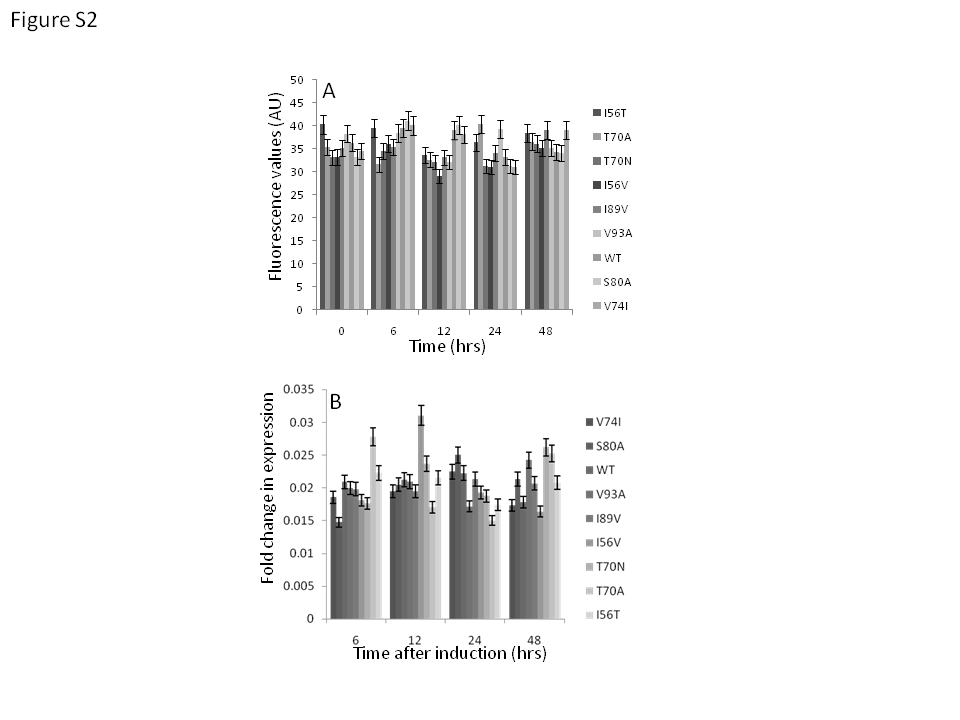

Supplement: Figure S2 — Expression from the action gene and PGK1 during HuL expression. A Transcript levels of actin during the expression of the HuL variants. Each bar represents the mean of 3 independent experiments and the error bars represent the standard deviation. B Gene expression levels of PGK1 during expression of the HuL variants. All data were normalised to the level of actin transcription and each bar represents the mean of 3 independent experiments with the standard deviation shown as error bars. Expression levels were measured by qRT-PCR and given as artificial units of fluorescence (AU). (TIF) [file pone.0022692.s002.tif]
