# Supplementary material for: Native-State Stability Determines the Extent of Degradation Relative to Secretion of Protein Variants from Pichia pastoris
Source: PLoS One. 2011 Jul 27;6(7):e22692. doi: 10.1371/journal.pone.0022692 (PMC3144928; doi:10.1371/journal.pone.0022692)
Supplement: Table S1 — Primers and probes. (DOC) [file pone.0022692.s003.doc]

| KAR2FP | TGGTATTCCACCAGCTCCAAG |
| --- | --- |
| KAR2RP | CAGTTCCCTTATCGGTGGCA |
| KAR2PROBE | TTACTTTTGTTTTAGACGCTAACGGAATTTTGAAGGTC |
| HAC1FP: | TTCGAAATCGGAGAAAGCAT |
| HAC1RP: | TTCGTCCTCTTCTTCGTCGT |
| HAC1PROBE | CATCCACTCGCAGATCTTTG |
| PDI1FP | CATTGGCTGAGTTTGTAAAGGAGA |
| PDI1RP | TTTTCTTCCTCGACTGGTCTGAG |
| PDI1PROBE: | AGGAACCCACAAGTGGATGCCCTAG |
| ActinFP | GTCCAGCATAAACACGCCG |
| ActinRP | CAGTGGGAAAAACCCACGAA |
| ActinPROBE | TGTGATGGAACAATGGTACTGGTCTTACCAAAC |
| MAL10FRAMFP | GGCGGCGGATCTGACAT |
| MAL10FRAMRP | CAGATGCAGACAGGGTGGAA |
| MAL10FRAMPROBE | CAGATGACCCAGTCTC |
| MAL10LINKFP | GGCTGAAGACCTGGCAGTTTA |
| MAL10LINKRP | CCGAACGTGAGCGGAGTACT |
| MAL10LINKPROBE | TACTGTCAGCAACATTC |
| MAL10FP | GCCTGGGGCTGAACTG |
| MAL10RP | TACTGGATACACTGGGTG |
| MAL10PROBE | TGCAAGGCTTCTGGCTACACC |

**Table S1:** Primers and probes (5’-3’).
